# Au/Ir@Zn/Cu-MOF-based lateral flow immunoassay employing high-affinity monoclonal antibody for sulfamethoxazole detection

**DOI:** 10.1016/j.fochx.2026.103525

**Published:** 2026-01-12

**Authors:** Linfang Lu, Lihong Wang, Kang Jiang, Na Li, Xiaoli Li, Zhoujie Xu, Shaoze Wu, Lakshani Madushika, Jun Yuan, Sumei Ling, Shihua Wang

**Affiliations:** The Ministry of Education Key Laboratory of Biopesticide and Chemical Biology, Fujian Key Laboratory of Pathogenic Fungi and Mycotoxins, School of Animal Science, School of Life Sciences, Fujian Agriculture and Forestry University, Fuzhou 350002, China

**Keywords:** Sulfamethoxazole, Monoclonal antibody, Molecular dynamics simulation, Lateral flow immunoassay, Metal-organic frameworks, Food safety

## Abstract

Sulfamethoxazole (SMX) is a widely used sulfonamide antibiotic, and its residues in animal-derived foods pose significant health risks. In this study, a high-affinity monoclonal antibody (2.65 × 10^10^ L/mol) against SMX was developed and used to construct an Au/Ir@Zn/Cu-MOF-based lateral flow immunoassay (LFIA). The probe was thoroughly characterized with good stability. The LFIA achieved a visual detection limit of 2.5 μg/mL, with an IC_50_ of 565.84 ng/mL and a linear detection range of 6.8 ng/mL-47.06 μg/mL. No cross-reactivity to structural analogues or matrix interference was observed in real food samples. Recovery rates (97.92–108%) were consistent with LC-MS results, confirming the accuracy of the method. This LFIA provides a sensitive and reliable tool for rapid SMX screening in food products.

## Highlights

• High-affinity anti-SMX monoclonal antibody (2.65 × 10^10^ L/mol).
• Au/Ir@Zn/Cu-MOF-based LFIA: LOD 6.8 ng/mL; IC_50_ of 565.84 ng/mL.
• High specificity with minimal matrix effects; 94.37–106.32% recoveries (LC-MS-verified).

## 1 Introduction

Sulfamethoxazole (SMX), a synthetic sulfonamide antibiotic, is widely used in veterinary medicine and aquaculture for its broad spectrum antibacterial activity and low production cost (Frigoli et al., 2025; Kennes-Veiga et al., 2022; Wu et al., 2024). However, excessive or improper use of SMX can result in its accumulation in animal derived food products, posing serious threats to human health, including allergic reactions (Gruchalla & Sullivan, 1991; Wu et al., 2024), antibiotic resistance (Singh et al., 2024; Thiebault, 2020), neurotoxicity (Wang et al., 2022; Y. Xu et al., 2022), and potential disruption of gut microbiota (Fan et al., 2024; Pei et al., 2024; Xiang et al., 2019). SMX is classified as a class III carcinogen (Hai et al., 2020), and regulatory authorities in many countries have set maximum residue limits (MRLs) for SMX in food. For instance, the European Union stipulates an MRL of 100 μg/kg in edible tissues (Frigoli et al., 2025). Therefore, developing rapid, sensitive, and reliable methods to detect SMX residues is essential for ensuring food safety and public health.

Currently, liquid chromatography-mass spectrometry (LC-MS) is considered the gold standard for SMX detection due to its high sensitivity and accuracy (Dubreil-Chéneau et al., 2014; K'Oreje et al., 2012). Alternative methods such as enzyme-linked immunosorbent assays (ELISA) (Krall et al., 2018), surface-enhanced Raman scattering (Patze et al., 2017), electrochemical sensors (Hu et al., 2021; Ramya et al., 2023), molecularly imprinted polymers (Karim et al., 2022), and solid phase extraction spectroscopy (Li et al., 2020) can provide faster detection but are often limited by multi-step operations, complex material synthesis, sophisticated instrumentation, or the need for laboratory settings.

**Scheme 1:**
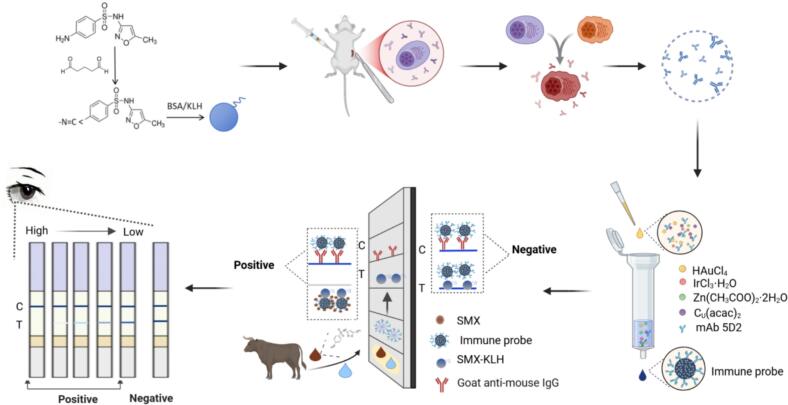
Preparation of anti-SMX antibody and establishment of immune detection based on Au/Ir@Zn/Cu-MOF.

Lateral flow immunoassays offer a rapid, low-cost, and user-friendly detection platform that is particularly suitable for on-site applications. Their performance, however, is largely dependent on the quality of the antibodies and the signal labels used. In this study, we developed a high-affinity monoclonal antibody (mAb) against SMX and engineered a multifunctional Au/Ir@Zn/Cu-MOF nanoprobe. This hybrid integrates the ocalized surface plasmon resonance and peroxidase-like activity of Au/Ir nanoparticles for enhanced colorimetric signal generation and amplification. Simultaneously, the porous Metal-organic frameworks (MOFs) scaffold facilitates high-density antibody immobilization and prevents nanoparticle aggregation, effectively preserving catalytic efficiency and ensuring exceptional colloidal stability (Furukawa et al., 2013). By integrating these features into the LFIA platform, we established a sensitive, robust, and field-deployable method for SMX detection (Scheme 1), offering strong potential for routine monitoring of antibiotic residues and improving food safety surveillanc.

## 2 Materials and methods

### 2.1 Materials

Sulfamethoxazole (SMX), bovine serum albumin (BSA), keyhole limpet hemocyanin (KLH), PEG 1450, HAT, HT, polyvinyl pyrrolidone (PVP), iridium (III) chloride hydrate (IrCl_3_·xH_2_O), zinc acetate dihydrate (Zn (CH_3_COO)_2_·2H_2_O), copper (II) acetylacetonate (Cu (acac)_2_) and imidazolate-2-carboxyaldehyde (2-ICA) were purchased from Sigma-Aldrich (St Louis, MO, USA). Dulbecco's modified eagle medium, fetal bovine serum, and antibiotics were obtained from Shenggong Bioengineering Co., Ltd. (Shanghai, China). Liquid paraffin, trisodium citrate dihydrate (C_6_H_5_Na_3_O_7_), CH_3_OH, dichloromethane, and chloroauric acid (HAuCl₄) were from China National Pharmaceutical Group Corporation (Beijing, China). *N*, *N*-dimethylformamide (DMF), sulfathiazole (ST), sulfamerazine (SMZ), sulfadimethoxine (SDM), and sulfamethazine (SM2) were sourced from Aladdin (Shanghai, China). Dicyclohexylcarbodiimide and glutaraldehyde were purchased from Macklin (Shanghai, China).

### 2.2 Preparation of the complete antigen

Complete antigens were produced by the glutaraldehyde method to couple the hapten to carrier proteins (BSA and KLH) (Sheng et al., 2006; Strijewski & Tang, 1990). Firstly, SMX (62.1 mg) and BSA (10 mg) were dissolved in 2.5 mL of phosphate-buffered saline (PBS, pH 7.4, 0.01 M), with stirring at 4 °C. Subsequently, 3 μL of 50% glutaraldehyde solution was added dropwise slowly. The reaction mixture was then transferred to room temperature and stirred continuously for 3 h in the dark. After the 3-h reaction period, the pH of the solution was adjusted to 9.0 using 0.1 M NaOH. The mixture was transferred to dialysis tubing (molecular weight cutoff: 10 kDa) and dialyzed extensively against 0.01 M PBS (pH 7.4) at 4 °C for 72 h to remove unreacted SMX, excess glutaraldehyde, and reaction by-products. The resulting conjugates were characterized by gel electrophoresis under denaturing conditions and UV–Vis spectroscopy. The dialyzed solution yielded the purified complete antigen SMX-BSA. The preparation method of complete antigen SMX-KLH is the same as that of SMX-BSA, except that BSA is replaced with KLH.

### 2.3 Preparation and characterization of anti-SMX monoclonal antibodies

The procedure for antibodies preparation followed protocols established in a prior study (Yin et al., 2023). BALB/c mice were immunized intraperitoneally with SMX-BSA conjugate emulsified in Freund's adjuvant. After four booster injections, serum antibody titers were monitored using indirect ELISA (iELISA), and the mouse with the highest response was selected for cell fusion. Splenocytes from the selected mouse were fused with SP2/0 myeloma cells using polyethylene glycol (PEG). Hybridoma cells were cultured in HAT selective medium and screened by iELISA for the secretion of anti-SMX antibodies. Positive clones were subcloned by limiting dilution until monoclonality was achieved. Chromosomal analysis was performed to confirm hybrid cell status, and antibody isotyping was conducted using a commercial mouse monoclonal antibody isotyping kit (PK20002, Proteintech). To produce ascitic fluid, monoclonal hybridoma cells were injected into the peritoneal cavity of liquid paraffin-pretreated BALB/c mice. Ascites were collected, and monoclonal antibodies (mAbs) were purified using Protein G affinity chromatography. The antibody titer, affinity, and cross-reactivity were evaluated by iELISA.

### 2.4 Molecular docking and molecular dynamics simulation

The three-dimensional structure of SMX-mAb was obtained by homology modeling based on its gene sequence (Ma et al., 2008). Sequence alignment was performed using BLAST, and the structural models were generated using MODELLER (Lee et al., 2022). The 3D structure of the small molecule SMX was constructed using RD Kit (Kunnakkattu et al., 2023). Molecular docking was carried out using AutoDock with a global search strategy, and the conformation with the lowest binding energy was selected for further analysis (Trott & Olson, 2009).

Molecular dynamics (MD) simulations were performed using GROMACS 2022.5 (Yu et al., 2024). The topology file of the ligand was generated using Sobtop 1.0 (dev3.1), while the topology of the antibody was built using the AMBER99SB force field. The complex was solvated in a cubic box filled with TIP3P water molecules, with a minimum distance of 1.0 nm between the protein surface and the box edges in all directions. To neutralize the system, Na^+^ and Cl^−^ ions were added as needed. Prior to the production run, energy minimization was carried out using the steepest descent algorithm for 2000 steps, followed by 100 ps equilibration under both NVT and NPT ensembles. The final MD simulations were conducted for 100 ns at 310 K and 1.0 bar under periodic boundary conditions. The integration time step was set to 2 fs, and the cutoff distances for Coulomb and van der Waals interactions were both set to 1.4 nm.

### 2.5 Synthesis of Au/Ir nanoparticles (Au/Ir NPs)

The method was a modified version of the synthetic method described in prior research (Wei et al., 2022; X. Xu et al., 2024). Au/Ir nanoparticles were synthesized via a modified citrate reduction approach. In brief, 193 mL of deionized water was mixed with 2 mL of 10% C_6_H_5_Na_3_O_7_ in a sterile conical flask and brought to a boil under constant stirring. Then, 1 mL of 1% HAuCl₄ solution and 4 mL of 1% IrCl_3_·xH_2_O were rapidly injected into the boiling mixture. The color transition from colorless to deep blue indicated the formation of Au/Ir NPs. The solution was maintained at boiling temperature for 1 h, cooled naturally to room temperature, and centrifuged at 12,000 rpm/min for 30 min at 25 °C. After discarding the supernatant, the nanoparticles were washed three times with deionized water and finally resuspended in 40 mL of ddH₂O for storage at 4 °C.

### 2.6 Fabrication of Au/Ir@Zn/Cu-MOF composite

Building on previous protocols, the synthesis of Au/Ir@Zn/Cu-MOF was optimized as follows (Stolar et al., 2021; Zhong et al., 2023). The metal-organic framework (MOF) encapsulating Au/Ir NPs was synthesized through a coordination-driven self-assembly process. Zn (CH_3_COO)_2_·2H_2_O (20.26 mg), Cu (acac)_2_ (10.22 mg), and PVP (60 mg) were dissolved in 35 mL of DMF and allowed to stand for 20 min. Subsequently, 20 mL of pre-synthesized Au/Ir NPs was added to the mixture. In parallel, 2-ICA (6.69 mg) was dissolved in 15 mL of DMF and slowly added dropwise. The resulting mixture was incubated at room temperature in the dark for 2 h. The final product was collected by centrifugation at 8000 rpm for 5 min (25 °C), washed thrice with ddH₂O, and resuspended in 50 mL of water. The Au/Ir@Zn/Cu-MOF composite was stored at 4 °C prior to use.

### 2.7 Conjugation of anti-SMX mAb to Au/Ir@Zn/Cu-MOF

To construct the immunoprobe, the Au/Ir@Zn/Cu-MOF nanomaterial was functionalized with anti-SMX monoclonal antibodies. A 10 mL aliquot of the MOF suspension was adjusted to mildly alkaline conditions by adding 50 μL of 0.1 M potassium carbonate, followed by the addition of 150 μL of purified SMX monoclonal antibody. After 60 min of gentle agitation at room temperature, 1% BSA was added to passivate the uncoated surface and reduce nonspecific adsorption. The reaction continued for another 30 min with 0.5% PEG 20,000 added as a stabilizer. After an additional 30 min of incubation, the conjugates were kept at 4 °C overnight to ensure complete interaction. The final probes were purified by centrifugation (12,000 rpm, 4 °C, 20 min) and was stored at 4 °C (Chen et al., 2025).

### 2.8 Assembly of Au/Ir@Zn/Cu-MOF immunochromatographic strip for SMX detection

To construct the lateral flow immunoassay (LFIA) device, a nitrocellulose (NC) membrane was mounted onto a PVC backing card (Bao et al., 2022). Using an automatic dispenser, the test line (T line) containing SMX-BSA conjugate and the control line (C line) coated with goat anti-mouse IgG were printed onto the NC membrane at defined positions. After drying, the Au/Ir@Zn/Cu-MOF-mAb were dispensed onto the conjugate pad. The sample pad, conjugate pad, NC membrane, absorbent pad, and PVC baseplate were sequentially laminated to form the complete LFIA strip. The assembled strips were cut into 4-mm widths and stored in sealed pouches with desiccant at room temperature.

### 2.9 Evaluation of sensitivity and selectivity of the developed LFIA

The analytical performance of the SMX-targeted LFIA was evaluated by preparing a dilution series of SMX standard solutions (Ouyang et al., 2022). After applying the samples onto the test strips, signal intensities at the T and C lines were recorded using a membrane reader. The inhibition curve was constructed by plotting the SMX concentrations (x-axis) against the normalized signal ratio (B/B₀, y-axis), where B₀ denotes the T/C ratio for the blank sample and B represents the ratio for each SMX concentration. The limit of detection (LOD) was defined as the analyte concentration corresponding to 90% inhibition. Cross-reactivity was assessed by testing structurally related sulfonamides and common veterinary drugs, including SMZ, ST, SM2, and SMD.

### 2.10 Real sample analysis and method verification

Six animal-derived foods (beef, pork, egg, eel, sea bass, and milk) were prepared as described (EDTA-McIlvaine extraction, vortexing, ultrasonication, centrifugation, pH adjustment, and 0.45 μm filtration) and diluted to the LFIA working range prior to analysis (Liu et al., 2025). Matrix effects were assessed by preparing blank extracts for each matrix, spiking them with SMX post-extraction, and constructing matrix-matched calibration curves alongside buffer-based curves. To minimize matrix interference, extracts were serially diluted (2×, 5×, 10×) with running buffer, and the optimal dilution or matrix-matched calibration was applied for quantification.

Recovery experiments were conducted by spiking SMX-free samples with known concentrations of SMX to assess the accuracy and precision of the method. To further validate the reliability of the LFIA, selected samples (egg, eel and beef) were analyzed in parallel using liquid chromatography-mass spectrometry (LC-MS) (Huang et al., 2025) for comparing the consistency.

### 2.11 Statistical analysis

All experimental data were derived from at least three independent replicates. Quantitative results for antibody titers and immunoassay responses were obtained by ELISA and strip reader analysis, respectively. Statistical processing and graphical presentation were carried out using Microsoft Excel, OriginPro 2023, GraphPad Prism 9, and BioRender.

## 3 Results and discussion

### 3.1 Synthesis and characterization of complete antigens

**Fig. 1:**
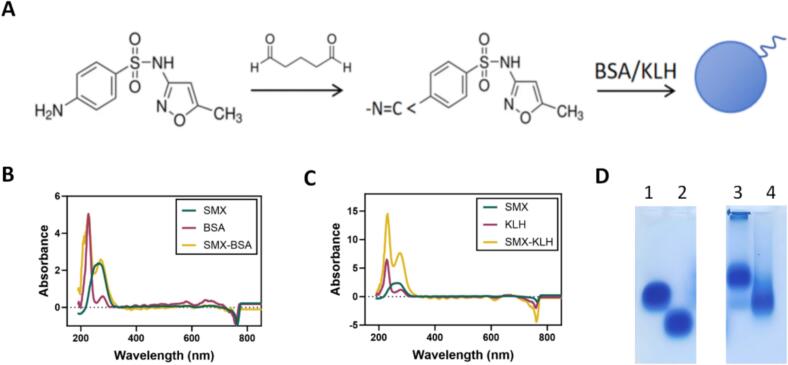
**Synthesis and evaluation of the complete antigens. (A)** The synthetic pathway of modified SMX haptens. **(B)** Characterization of the complete antigen SMX-BSA by UV–Vis spectroscopy. **(C)** Characterization of the complete antigen SMX-KLH by UV–Vis spectroscopy. **(D)** Analysis of conjugates by agarose gel electrophoresis. Lane 1: BSA. Lane 2: SMX-BSA. Lane 3: KLH. Lane 4: SMX-KLH.

The design of hapten structures is critical in antibody production, as it significantly affects the sensitivity and specificity of the resulting antibodies, as well as the competitive efficiency of the coating antigens. SMX contains an amino group, which allowing covalent conjugation to macromolecular carrier proteins. In this study, SMX was coupled to BSA and KLH via the glutaraldehyde method, utilizing nucleophilic addition between aldehyde and amino groups (Fig. 1A). The successful synthesis of the complete antigens, SMX-BSA (immunogen) and SMX-KLH (coating antigen), was verified by UV–visible spectroscopy. As shown in Fig. 1**B** and **C**, characteristic shifts in the absorption peaks were observed. In addition, agarose gel electrophoresis was employed to further evaluate the conjugates. The migration rate of the conjugated products was faster than that of the carrier proteins alone (Fig. 1D), suggesting an increased net charge and confirming successful conjugation of the complete antigens.

### 3.2 Preparation and characterization of monoclonal antibodies against SMX

**Fig. 2:**
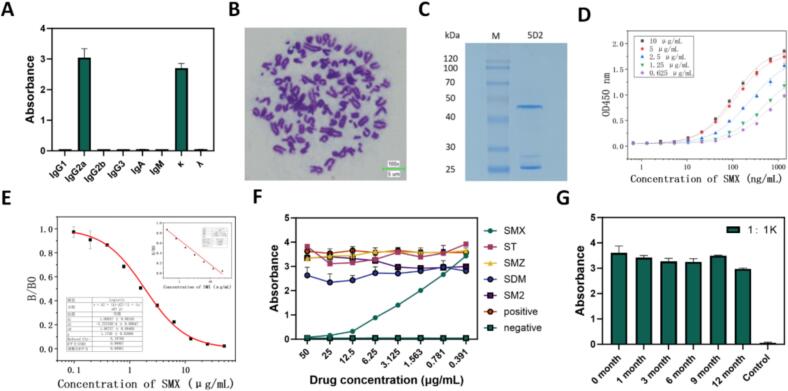
**Characterization of antibodies against SMX. (A)** Sub-type identification of 5D2 mAb. **(B)** Chromosome analysis of the 5D2 cell line. **(C)** SDS-PAGE analysis of purified antibodies secreted by the 5D2 cell line. **(D)** Affinity analysis of the anti-SMX mAb secreted by the 5D2 cell line. **(E)** Standard curve and linear range of SMX-mAb for SMX detection in ELISA. **(F)** Cross-reactivity of 5D2 mAb with similars. **(G)** Titer analysis of Anti-SMX mAb diluted 1000 times at different time points.

To generate SMX-specific immunoreagents, female BALB/c mice were immunized with SMX-BSA emulsified in Freund's adjuvant following a primary-boost schedule at 14-day intervals. Indirect ELISA (iELISA) analysis revealed that the mice produced a strong immune response after the fourth immunization, with an antibody titer reaching 1.6 × 10^4^. Following cell fusion between splenocytes and SP2/0 myeloma cells, a positive hybridoma clone named 5D2 was obtained through multiple rounds of subcloning. Isotyping of 5D2 revealed that the monoclonal antibody belonged to the IgG2a subclass with a kappa light chain (Fig. 2A). Chromosome analysis confirmed successful cell fusion, showing that the 5D2 cell line possessed 106 ± 3 chromosomes (Fig. 2B). The positive hybridoma cells were injected intraperitoneally into the peritoneal cavity of mice, and ascites fluid was collected from the mice, then the monoclonal antibody (SMX-mAb) was purified using Protein G affinity chromatography. SDS-PAGE analysis showed distinct bands corresponding to the antibody heavy (50 kDa) and light chains (25 kDa) (Fig. 2C), indicating successful purification. The purified antibody exhibited a high titer exceeding 8 × 10^3^. Notably, the affinity constant of SMX-mAb was determined to be 2.65 × 10^10^ L/mol (Fig. 2D), confirming that SMX-mAb was a high-affinity monoclonal antibody suitable for immunoassay development. Under optimal concentration of the antibody and SMX, the IC_50_ values of icELISA was 1.867 μg/mL (Fig. 2E), indicating that SMX-mAb can be directly applied to ELISA-based assay formats and has potential for integration into ELISA or other immnoassay kits.

Cross-reactivity is a key concern when developing immunoassays for small molecules. The specificity of SMX-mAb was assessed by icELISA using four structurally related sulfonamides: ST, SMZ, SDM, and SM2. As shown in Fig. 2F, no detectable cross-reactivity was observed, demonstrating the excellent specificity of the antibody. To evaluate long-term stability and support practical translation, SMX-mAb was further subjected to a one-year stability assessment. As shown in Fig. 2G, the antibody maintained excellent stability throughout the 12-month follow-up. Notably, even after a 1:1000 dilution, the ELISA signal remained strong, with an OD450 nm value exceeding 3, indicating minimal loss of immunoreactivity over time. This robust long-term stability is advantageous for large-scale production and routine immunoassay applications.

### 3.3 Molecular docking and dynamics simulation results

**Fig. 3:**
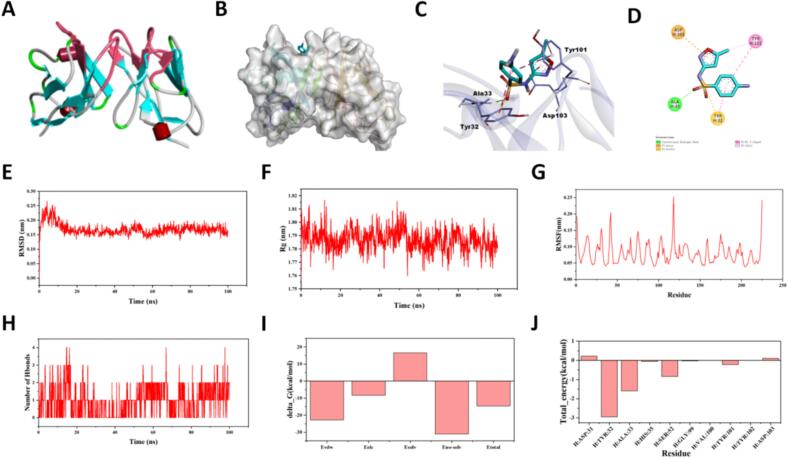
**Molecular docking and molecular dynamics simulation of the SMX-antibody complex. (A)** Antibody homology modeling structure. **(B)**Binding pose of SMX within the heavy chain CDR region of the antibody. **(C)** Hydrogen bond formed between SMX and the backbone of Ala33. **(D)** Hydrophobic and π-anion interactions involving Tyr32, Tyr101, and Asp103. **(E)** RMSD trajectory of the antibody backbone over 100 ns. **(F)** Radius of gyration (Rg) profile. **(G)** RMSF values. **(H)** Dynamic changes in the number of hydrogen bonds between SMX and the antibody. **(I)** MM-PBSA-calculated binding free energy. **(J)** Per-residue energy decomposition.

Molecular docking results revealed that SMX binds to the complementarity-determining region (CDR) of the antibody heavy chain (Fig. 3A). Specifically, SMX formed a hydrogen bond with Ala33, hydrophobic interactions with Tyr32, Tyr101, and Asp103, as well as a π–anion interaction with Asp103 (Fig. 3B-3D). During the 100-ns molecular dynamics (MD) simulation, the root-mean-square deviation (RMSD) gradually increased and stabilized after approximately 58 ns, indicating that the system had reached equilibrium (Fig. 3E). The radius of gyration (Rg) showed minor fluctuations, suggesting local conformational adjustments without loss of global structural integrity (Fig. 3F). Root-mean-square fluctuation (RMSF) analysis demonstrated greater flexibility in the surface loop regions (Fig. 3G). Hydrogen bond analysis revealed dynamic fluctuations, with an average of three hydrogen bonds maintained between SMX and the antibody in the latter stages of the simulation (Fig. 3H). Molecular mechanics Poisson-Boltzmann surface area (MM-PBSA) analysis estimated a binding free energy of −14.56 ± 1.09 kcal/mol (Fig. 3I). Energy decomposition indicated that Tyr32 contributed the most significantly (−2.95 kcal/mol) to ligand binding (Fig. 3J). Collectively, these results suggest a stable binding interaction primarily driven by hydrophobic contacts and limited hydrogen bonding, with structural equilibrium achieved during the simulation.

### 3.4 Preparation of the Au/Ir@Zn/Cu-MOF probes

**Fig. 4:**
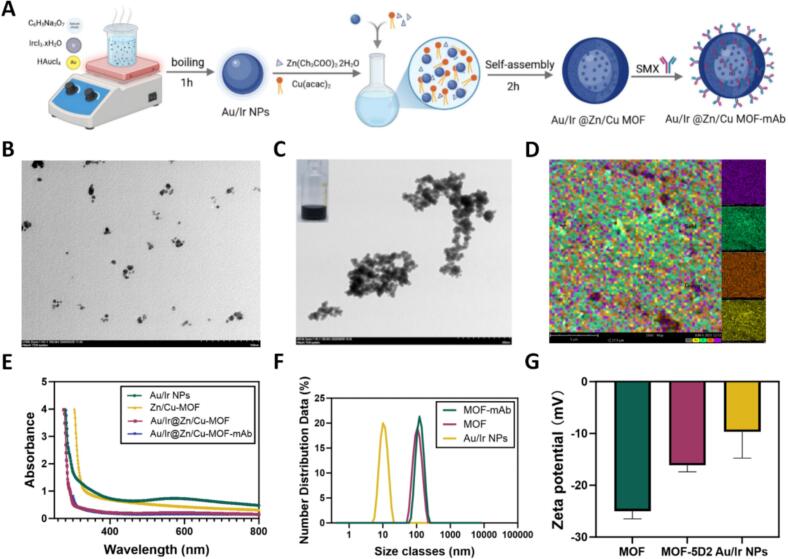
**Characterization of the Au/Ir@Zn/Cu-MOF probe. (A)** The preparation process of the Au/Ir@Zn/Cu-MOF-mAb. **(B)** TEM images of Au/Ir NPs. **(C)** TEM images of the Au/Ir@Zn/Cu-MOF. **(D)** EDS of the Au/Ir@Zn/Cu-MOF. **(E)** The change in the UV–Vis spectrum of Au/Ir NPs, the Zn/Cu-MOF, the Au/Ir@Zn/Cu-MOF, and the Au/Ir@Zn/Cu-MOF-mAb. **(F)** The change of particle size of Au/Ir NPs, the Au/Ir@Zn/Cu-MOF, and the Au/Ir@Zn/Cu-MOF-mAb detected by DLS. **(G)** The change of Zeta potential of Au/Ir NPs, the Au/Ir@Zn/Cu-MOF, and the Au/Ir@Zn/Cu-MOF-mAb.

The Au/Ir@Zn/Cu-MOF nanocomposite was synthesized using a co-precipitation method (Fig. 4A). Transmission electron microscopy (TEM) analysis revealed that Au/Ir nanoparticles (NPs) were uniformly embedded within the Zn/Cu-MOF structure, demonstrating distinct morphological differences between Au/Ir NPs and the final Au/Ir@Zn/Cu-MOF composite (Fig. 4B, C). Subsequently, the synthesized Au/Ir@Zn/Cu-MOF was conjugated with the 5D2 monoclonal antibody to form the immunoprobe. UV–visible spectra recorded from 300 to 800 nm confirmed the conjugation, and energy-dispersive X-ray spectroscopy (EDS) analysis verified the presence of Au, Ir, Zn, and Cu elements in the final composite (Fig. 4D). The optical properties of Au/Ir NPs, Zn/Cu-MOF, Au/Ir@Zn/Cu-MOF, and Au/Ir@Zn/Cu-MOF-mAbs were compared via UV–vis spectroscopy (Fig. 4E). A progressive red shift in the absorption peaks was observed in the order: Au/Ir NPs > Zn/Cu-MOF > Au/Ir@Zn/Cu-MOF > Au/Ir@Zn/Cu-MOF-mAbs, indicating successful surface modification and antibody conjugation. Dynamic light scattering (DLS) analysis showed an increase in hydrodynamic diameter from 10.10 nm (Au/Ir NPs) to 105.71 nm (Au/Ir@Zn/Cu-MOF), and further to 122.42 nm after antibody coupling (Fig. 4F), consistent with successful probe complex formation. Zeta potential analysis provided additional confirmation of conjugation success, showing a shift from −9.7 mV (Au/Ir NPs) and − 25.01 mV (Au/Ir@Zn/Cu-MOF) to −16.17 mV for the final Au/Ir@Zn/Cu-MOF-mAbs probe (Fig. 4G). Collectively, these results confirmed the successful fabrication and functionalization of the Au/Ir@Zn/Cu-MOF immunoprobe for SMX detection.

### 3.5 Construction and evaluation of the Au/Ir@Zn/Cu-MOF-based immunoassay

**Fig. 5:**
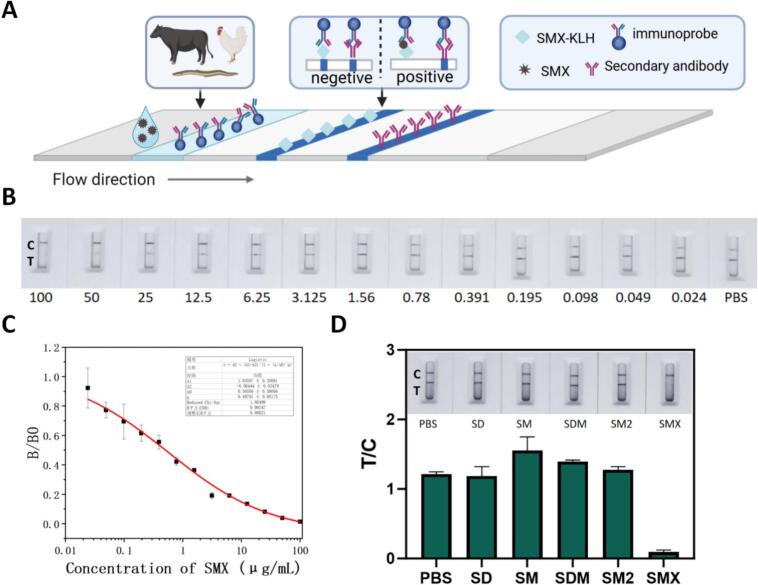
**Evaluation of the Au/Ir@Zn/Cu-MOF based LFIA. (A)** Work schematic draw of LFIA. **(B)** Sensitivity of the Au/Ir@Zn/Cu-MOF-based test strip determined by testing different concentrations of SMX (μg/mL) by the naked eye. **(C)** Standard curve and linear range of the SMX-mAb for SMX detection using the Au/Ir@Zn/Cu-MOF. **(D)** Evaluation of the specificity of the Au/Ir@Zn/Cu-MOF-based LFIA.

As shown in Fig. 5A, the LFIA works on a competitive principle: SMX in the sample competes with SMX-BSA on the test line for the Au/Ir@Zn/Cu-MOF-labeled antibody. The control line, coated with goat anti-mouse IgG, binds excess probes to confirm the valid. Higher SMX levels produce weaker color on the test line. To optimize the performance of the Au/Ir@Zn/Cu-MOF-mAb-based lateral flow immunoassay (LFIA), critical parameters including the concentration of goat anti-mouse IgG on the control line (C line), the coating antigen on the test line (T line), and the amount of labeled antibody probe, were systematically investigated. The optimal conditions were identified as follows: 1 mg/mL goat anti-mouse IgG for the C-line, 150 μg/mL coating antigen for the T-line, and 2 μL probe volume per strip. Under these optimized conditions, the Au/Ir@Zn/Cu-MOF-mAb-based LFIA was developed for SMX detection.

To evaluate assay sensitivity, SMX standards ranging from 100 μg/mL to 0.024 μg/mL were tested. As shown in Fig. 5B, the intensity of the T-line decreased with the increasing SMX concentration, and the disappearance of the T-line was visually observed at 50 μg/mL. Thus, the visual limit of detection (vLOD) for SMX was estimated to be 50 μg/mL. Furthermore, a quantitative standard curve was established based on the inhibition rate of the T-line signal intensity, achieving a strong linear correlation (R^2^ = 0.992), with an IC_50_ of 565.84 ng/mL and a linear detection range from 6.8 ng/mL to 47.06 μg/mL (Fig. 5C). To assess specificity, the assay was challenged with structurally related sulfonamides (ST, SMZ, SDM, and SM2) at a concentration of 50 μg/mL. As demonstrated in Fig. 5D, no detectable signal interference or cross-reactivity was observed, confirming that the Au/Ir@Zn/Cu-MOF-mAb-based LFIA exhibits specificity for SMX.

### 3.6 Stability, feasibility and practical application of the developed LFIA in food samples

**Fig. 6:**
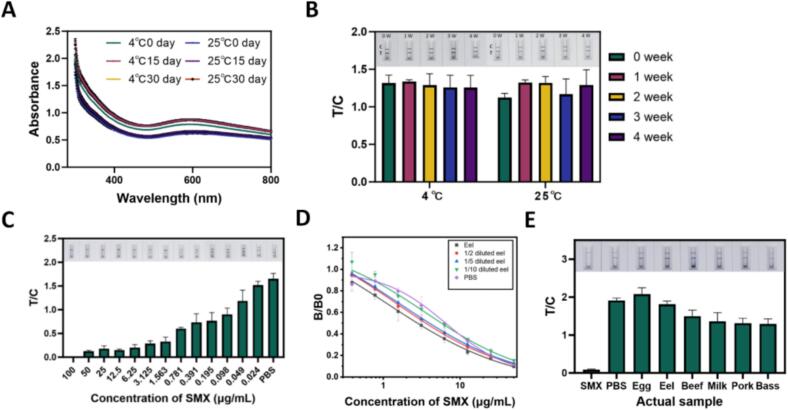
**Evaluation of stability, matrix effects, and practical applicability of the Au/Ir@Zn/Cu-MOF-based test strip. (A)** UV–Vis absorption spectra of the Au/Ir@Zn/Cu-MOF-mAb probe under various storage temperatures and time intervals. **(B)** Storage stability of the test strip evaluated using PBS. **(C)** Visual detection performance and consistency of the fabricated strips for SMX at varying concentrations after 30 days of storage. **(D)** Matrix interference analysis and standard curves in eel samples. **(E)** Practical application of the developed test strip for SMX detection in real food samples.

To address the stability requirement for practical use, the Au/Ir@Cu/Zn-MOF-mAb probe and the assembled LFIA strips were systematically evaluated during storage at 4 °Cand 25 °C(room temperature). Unless otherwise specified, the fabricated strips were stored in a sealed container with relative humidity (RH) maintained below 30%. As shown in Fig. 6A, the UV–vis spectra of Au/Ir@Cu/Zn-MOF-mAb exhibited negligible variation across temperatures and time points, indicating good physicochemical stability of the probe. Stability was further quantified using a membrane strip reader, and the optical density signals remained essentially unchanged over the storage period (Fig. 6B), supporting robust and reproducible readouts. In addition, the stability of the Au/Ir@Cu/Zn-MOF-based test strips was verified by repeatedly testing different concentrations of SMX (μg/mL) and visually inspecting line development. As presented in Fig. 6C, clear and consistent bands were observed by naked eye, demonstrating stable strip performance during storage. Collectively, the stability result indicates that both the probe and the LFIA strips remain stable under storage at 4 °Cand 25 °C (RH < 30%), with excellent storage stability of at least one month.

The feasibility of the Au/Ir@Zn/Cu-MOF-based LFIA for SMX detection was evaluated through analyses of real food samples and spiked recovery experiments. Six common food matrices were tested, including beef, pork, eel, sea bass, milk, and egg. To evaluate potential matrix effects, one representative samples were selected. As shown in Fig. 6**D**, no significant signal interference was observed, indicating minimal matrix impact. Samples spiked with 50 μg/mL SMX were used as the positive control, while PBS served as the negative control. Visual inspection revealed a complete disappearance of the T-line at 50 μg/mL SMX, confirming the previously determined visual detection limit. Quantitative analysis using a strip reader demonstrated clear differences in T/C ratios between untreated and spiked samples. As illustrated in Fig. 6E, unspiked samples exhibited high T/C values, whereas spiked samples showed significantly lower values, demonstrating the assay's accuracy and sensitivity. Importantly, no SMX residues were detected in any of the commercial food samples tested. To further assess accuracy, recovery studies were conducted and compared with the results obtained from LC-MS analysis. As summarized in Table 1, the LFIA achieved recovery rates of 97.92–108%, with coefficients of variation (CV) below 11.17%. These results closely matched the LC-MS results, confirming the reliability and accuracy of the developed LFIA for SMX detection in food samples.

**Table 1:** Summary of LFIA detection results for real samples.

Detection	Sample	Spiked concentration (μg/mL)	Measured concentration (μg/mL)	Recovery (%)	CV (%)
Au/Ir@Zn/Cu-MOF	Beef	50	50.09	100.18 ± 1.00	1.00
		3.125	3.06	97.92 ± 6.66	6.80
		0.1	0.11	108 ± 12.07	11.17
	Eel	50	51.42	102.84 ± 0.42	0.40
		3.125	3.12	99.84 ± 3.41	3.42
		0.1	0.10	100 ± 10.33	10.34
	Egg	50	51.42	102.84 ± 0.50	0.49
		3.125	3.15	100.8 ± 7.23	7.17
		0.1	0.10	98.4 ± 8.68	8.82
LC-MS	Beef	50	49.2	98.39 ± 0.57	0.58
		3.125	3.28	105.01 ± 0.52	0.50
		0.1	0.08	80.73 ± 0.47	0.58
	Eel	50	55.13	111.29 ± 0.24	0.22
		3.125	3.63	116.22 ± 0.13	0.11
		0.1	0.09	93.51 ± 0.83	0.89
	Egg	50	51.47	102.96 ± 0.91	0.89
		3.125	3.19	105.36 ± 0.67	0.63
		0.1	0.08	76.12 ± 0.29	0.38

Recovery = Measured concentration/spiked concentration × 100%.

CV = Standard deviation/mean × 100%.

## 4 Conclusion

In summary, a monoclonal antibody with high-affinity and specificity against SMX was successfully developed and utilized to construct a novel Au/Ir@Zn/Cu-MOF-based lateral flow immunoassay (LFIA). The established assay exhibited excellent sensitivity, achieving a detection limit of 6.8 ng/mL and IC_50_ of 565.84 ng/mL. The method demonstrated strong anti-interference ability in complex food matrices, and recovery rates closely matched those obtained by LC-MS, confirming the accuracy and reliability of the assay. These findings underscore the promising potential of the developed LFIA platform as a rapid and practical tool for on-site screening of SMX residues in food safety monitoring.

For euthanasia, animals were exposed to carbon dioxide until unconscious, followed by cervical dislocation to ensure humane termination. Animals were euthanized via a CO2 gradual-fill method (20–30% chamber volume/min) in a transparent chamber equipped with a precision flowmeter. Animals were observed continuously, and gas flow was maintained until loss of righting and pedal withdrawal reflexes, followed by ≥1 min after respiratory arrest. Chambers were loaded to avoid overcrowding, and procedures were conducted by trained personnel. Carcasses were promptly sealed in biohazard bags and disposed of by incineration in accordance with institutional guidelines and relevant EU regulations for the disposal of animal waste.

## CRediT authorship contribution statement

**Linfang Lu:** Writing – original draft, Methodology, Formal analysis, Data curation, Conceptualization. **Lihong Wang:** Methodology. **Kang Jiang:** Methodology, Data curation. **Na Li:** Data curation. **Xiaoli Li:** Validation. **Zhoujie Xu:** Software. **Shaoze Wu:** Methodology. **Lakshani Madushika:** Writing – review & editing. **Jun Yuan:** Conceptualization. **Sumei Ling:** Writing – review & editing, Conceptualization. **Shihua Wang:** Writing – review & editing, Supervision, Resources, Conceptualization.

## Ethical statement

All animal experiments were conducted in compliance with the Regulations for the Administration of Affairs Concerning Experimental Animals and were approved by the Ethics Committee of Fujian Agriculture and Forestry University, China (Approval No. PZCASFAFU24118). In addition, all procedures involving laboratory mice adhered to the European Directive 2010/63/EU on the protection of animals used for scientific purposes. Mice were maintained in a specific-pathogen-free (SPF) facility under controlled environmental conditions (22 ± 2 °C temperature, 55 ± 5% humidity, and a 12-h light/dark cycle), with unrestricted access to standard chow and water. Every effort was made to minimize animal suffering and distress throughout the experimental period.

## Declaration of competing interest

The authors declare that they have no known competing financial interests or personal relationships that could have appeared to influence the work reported in this paper.

## Data Availability

Data will be made available on request.
